# The beginning of connectomics: a commentary on White *et al.* (1986) ‘The structure of the nervous system of the nematode *Caenorhabditis elegans*’

**DOI:** 10.1098/rstb.2014.0309

**Published:** 2015-04-19

**Authors:** Scott W. Emmons

**Affiliations:** Department of Genetics, Albert Einstein College of Medicine, Bronx, NY, USA

**Keywords:** connectomics, *Caenorhabditis elegans*, nervous system

## Abstract

The article ‘Structure of the nervous system of the nematode *Caenorhabditis elegans*' (aka ‘The mind of a worm’) by White *et al.*, published for the first time the complete set of synaptic connections in the nervous system of an animal. The work was carried out as part of a programme to begin to understand how genes determine the structure of a nervous system and how a nervous system creates behaviour. It became a major stimulus to the field of *C. elegans* research, which has since contributed insights into all areas of biology. Twenty-six years elapsed before developments, notably more powerful computers, made new studies of this kind possible. It is hoped that one day knowledge of synaptic structure, the *connectome*, together with results of many other investigations, will lead to an understanding of the human brain. This commentary was written to celebrate the 350th anniversary of the journal *Philosophical Transactions of the Royal Society*.

## 1. A foundational publication

In August, 1984, two copies of a very large manuscript, each filling six loose-leaf notebooks, arrived at the Royal Society in London. They were accompanied by a cover letter from the senior author, Sydney Brenner, addressed to Prof. Brian Boycott, a neuroscientist known to Brenner who was an Associate Editor of the society's journal. The letter explained that the manuscript was being sent for publication and though the notebooks were labelled―perhaps whimsically, perhaps provocatively―‘The mind of a worm’, the correct title would be found in the notebook labelled ‘Text and figures’.

The landmark paper, ‘The structure of the nervous system of the nematode *Caenorhabditis elegans*' [1] described for the first time the map of the entire nervous system of an animal. It represented the completion of the initial phase of a project initiated over two decades before by South African-born Brenner, now based in Cambridge, England. The ambitious goal of this project was to begin to confront the problem of the brain, the body's most complex and enigmatic organ. During the 1950s and early 1960s, Brenner had made a major contribution to the revolutionary discoveries that revealed how genes replicate and convey information. Now he wanted to apply the same powerful genetic techniques he had used in this breakthrough work to understand the nervous system [2]. Brenner chose a tiny (1 mm), transparent nematode worm, an animal with only 1000 cells, as the centrepiece of this research. By 1984, the complete wiring diagram of the little worm's nervous system had been ascertained.

With their paper, Brenner and his co-workers founded the yet-to-be-named field of *connectomics*, the determination and study of complete maps of the synapses in nervous systems. Beyond this, Brenner achieved something rare in science: the establishment of an entire, wide-ranging field of scientific inquiry from the choice of material and problem by a single scientist. Discoveries made in the field of *C. elegans* research have ranged well beyond the nervous system into all corners of biology and have garnered three Nobel Prizes honouring eight scientists, including Brenner himself.

## 2. Selecting the worm

Brenner's laboratory was at the Medical Research Council Laboratory of Molecular Biology in Cambridge (MRC-LMB). When he first began to turn his attention to the nervous system, Brenner felt he needed to find a suitable experimental organism. Among current models, one popular, well-studied choice, the fruit fly *Drosophila melanogaster*, had good genetics and interesting behaviour but seemed too complex as its nervous system contained some 100 000 neurons. Others, such as the slime mould *Dictyostelium discoidium*, had no nervous system at all. A new model animal was needed.

In seeking a new experimental animal to tackle problems of the nervous system, Brenner was not alone. Two scientists at Columbia University, Cyrus Levinthal and Eduardo Macagno, were similarly exploring a number of possibilities [3]. However, Brenner was uniquely guided by an important insight: not only would it be necessary to select an animal with accessible genetics and some amount of interesting behaviour, it would also be necessary to find one whose nervous system could be described completely at the synaptic level.

The nervous system is a network of connected neurons. Its properties emerge in part from the pattern of these connections. Although it was clear a structural description would be insufficient—‘The behaviour of an organism is very remote from the elementary actions of genes and, even if simple paradigms analogous to the one gene–one enzyme rule exist, they may not be easy to find’ [4, p. 269]—nevertheless, a structure would be essential. Just as in the earlier work on the genetic code, which used bacteria and their viruses, genetic analysis alone would be insufficient to interpret the effects of mutations. Moreover, knowing the structure, Brenner pointed out, would allow the problem to be divided into two: how do genes specify the structure, and how does the nervous system create behaviour. The idea of structure was not new to neuroscience. From the time of the great Spanish neuroanatomist Santiago Ramón y Cajal in the late nineteenth century to the first half of the twentieth, Sherrington, de No, Adrian and many others sought to trace out anatomical circuits in the brain and spinal cord. What was new was to tackle the nervous system armed with a combination of behavioural mutants and a complete wiring diagram. Ten years after beginning to think about the problem, by 1973 Brenner described a well-defined programme: ‘Thus, what has to be done is clear in general outline: i.e. isolate mutants affecting behaviour of an animal and see what changes have been produced in the nervous system’ [4, p. 269].

The only way to determine the detailed synaptic structure of a nervous system was to use electron microscopy (EM). To trace neurons and their connections, cell membranes and synapses needed to be seen. These structures are below the resolving power of the light microscope. The idea was to reconstruct a nervous system from serial section electron micrographs, learning in the process how all the neurons were connected to each other via synapses, and thus produce the wiring diagram. This experimental approach was severely limiting because the observation window of the electron microscope is tiny and it would be necessary to image thousands of extremely thin sections. Brenner set about reading widely through the zoological literature to see if he could identify a millimetre-sized animal with the other two requisite properties—genetics and behaviour.

At this time, an experienced electron microscopist happened to be looking for a job. Nichol Thomson had been Lord Victor Rothschild's technician, but Rothschild was leaving research. Sydney and Victor shared martinis on weekends. Sydney hired Nichol to help in the survey of potential organisms. This was a lucky happenstance. Although Nichol did not have an advanced education (which caused difficulty in securing his appointment), it became clear that he was ‘a man of great skill’ [5]. For over 20 years until his retirement, Nichol provided many long unbroken series of ultrathin sections of specimens and electron micrographs of them.

**Figure 1.**
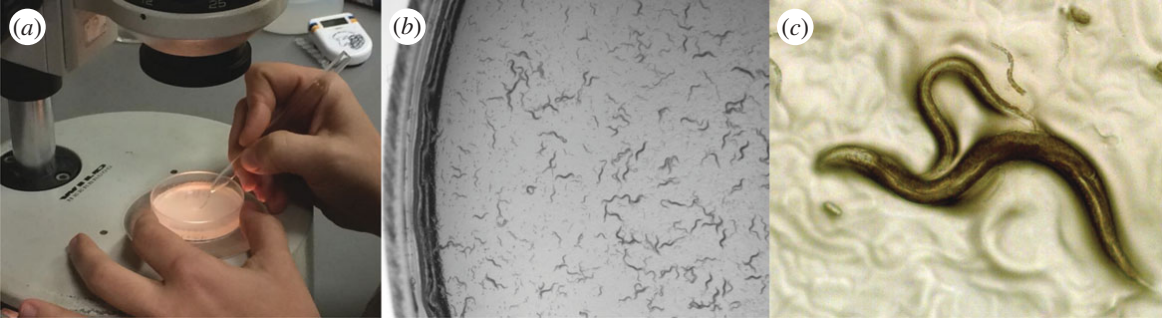
The free-living nematode *Caenorhabditis elegans*, 1 mm long, grows on an agar plate, feeding on a lawn of *Escherichia coli* bacteria. (*a*) An experimenter selects an individual worm with a platinum wire. (*b*) A developing population with adults, juveniles and eggs. (*c*) Two adult worms mating, along with larval worms and eggs. The two sexes are a male and a self-fertile hermaphrodite, which is a female that makes 300 of its own sperm. The male, the smaller adult worm, has the copulatory organ at its tail anchored at the hermaphrodite mid-body vulva. Its sperm will also fertilize the hermaphrodite's eggs. Images provided by the author.

**Figure 2.**
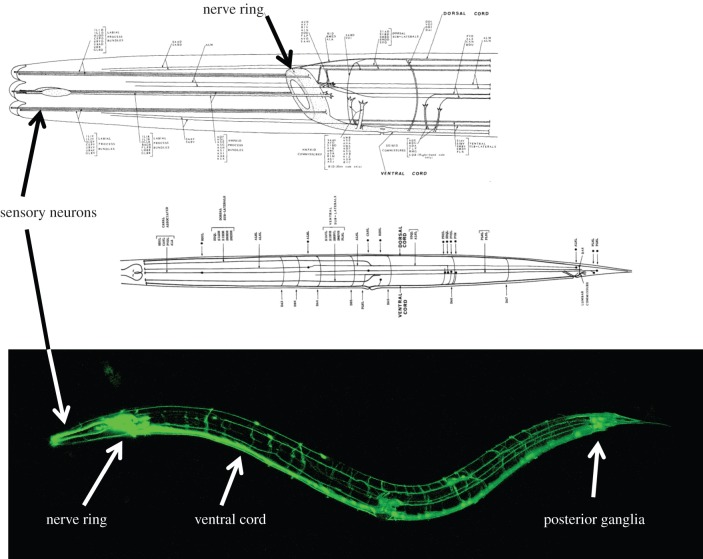
The *C. elegans* nervous system. Top: diagrams from ‘The mind of a worm’ [1, figs 6 and 7]. Bottom: a worm expressing the fluorescent protein GFP in its entire nervous system. The hermaphrodite nervous system contains precisely 302 neurons, the male, 383 (46% of its somatic nuclei). The nerve ring surrounding the pharynx contains complex circuitry governing most aspects of behaviour. This is the closest thing the worm has to a ‘brain’. The ventral nerve cord contains motorneurons that govern undulatory locomotion. Many sensory neurons have endings arrayed around the mouth. The extra male neurons are mostly situated in the tail where they form the circuits for mating. (Picture from Hang Ung, Jean-Louis Bessereau laboratory, France, with permission.)

In his search through zoology, Brenner would identify and obtain some potentially suitable tiny invertebrate, sometimes collecting it himself from local pond water, give it to Nichol to fix and section, and examine the images to see whether neuron membranes and synaptic structures could be seen. In 1963, he wrote to the Berkeley nematologist Ellsworth Dougherty and asked for a culture of the nematode *C. elegans*. Nichol's first attempt with *C. elegans* gave an unfavourable result. But this species met the other two requirements so well that Sydney asked him to please have another try. It transpired that the first animal examined had been a dauer larva, a larval form with an impenetrable cuticle adapted for survival. Fortunately, for the second attempt, an adult animal was selected and a clear image of the nervous system emerged. This species was chosen (figure 1). Its favourable features included a small number of neurons, around 300, identical cellular makeup in every individual, short life cycle (3½ days), easy rearing in the laboratory and good genetics (figure 2). It would live on the two-dimensional surface of an agar plate, making it easy to observe with a dissecting microscope.

## 3. Making the map

Obtaining large sets of decipherable electron micrographs was just the first step in obtaining a wiring diagram. Electron micrographs of tissues are extremely rich in detail. The cell membranes of thin neural processes had to be discerned along with their many synapses and discriminated from the myriad of other intra- and intercellular structures present. Synapses are of two kinds, chemical and electrical (gap junctions). Enumerating all the hundreds of relevant structures and tracing them through thousands of images was a daunting task.

Brenner thought the computer could be used for this annotation phase and in a second lucky hire, in 1969, he obtained the assistance of John White, who had just received his undergraduate degree in physics and was an autodidact in electronics and computers. White had experience in computer graphics programming. Brenner had already examined micrographs Nichol made of mutants and found that abnormalities in the nervous system could be seen. Now he passed the EM part of the project over to White while he focused his attention on isolating more behavioural mutants and establishing the methodology of genetic analysis of the worm [6].

**Figure 3.**
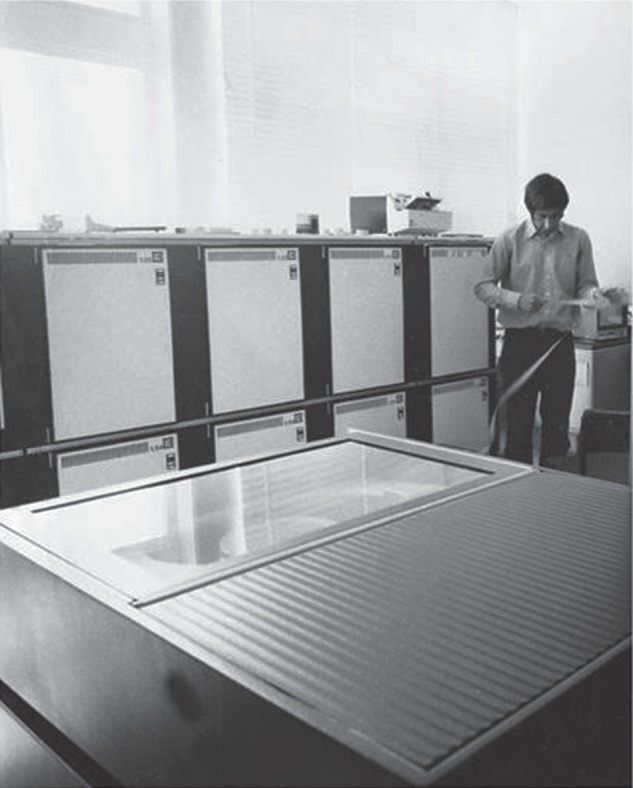
John White standing next to the Modular I computer. The box in front is the storage disk drive. Reproduced with permission from [7].

An advanced laboratory computer, a Modular I, was purchased (figure 3). Input to this machine was from punched paper tape. An operating system for it had to be written. Then a text editor, a disk filing system, and drivers for graphics displays. Most of this programming was done in assembly language. All this had to be done before programs specifically for nervous system reconstruction could be contemplated—the app in today's parlance. This turned out to be the easy part. A digitizing tablet was designed and constructed as a coordinates input device. A contraption was devised for aligning the electron micrographs and transferring the aligned images onto a filmstrip so that they could be back-projected one by one onto the tablet for tracing the cell outlines. By putting these outlines together through the stack of images, a three-dimensional reconstruction of the nervous system could be created. Eventually, a system based on all this was made to work [8]. John White wrote it up for his PhD thesis [9]. But it never worked well enough. The computer, though room-sized, had only 64 K of memory—or in today's terms, 0.000064 GB! The storage drive had a capacity of 22 MB. As tiny as the worm and its nervous system is, the job was still too big for the Modular I. In this effort, John and Sydney had the right idea but were a couple of decades ahead of their time.

**Figure 4.**
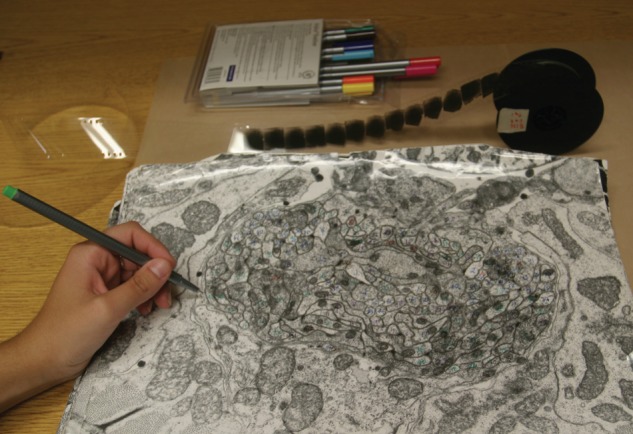
Large paper prints were marked with Rotring Rapidograph coloured pens to trace neurons through the stack of images. Movie film reel of serial EM images shown in the background. (MRC archives, Hall laboratory, Albert Einstein College of Medicine.)

In the end, the reconstruction had to be done by hand and technician Eileen Southgate became involved. Fortunately, she liked to work on puzzles at home. She generated electron micrographs from Nichol's series of sections and printed them as large, 12 × 16 inch glossy prints. Brenner had originally traced the images onto transparent overlays and marked the overlays with wax pencils or felt tip pens. But the pencils and pens were too large to mark the smallest processes and there were not enough colours to distinguish the different neurons. The discovery of Rotring Rapidograph pens was key. They had finer tips, many colours, and were erasable with alcohol. The prints could be marked directly with these pens, generating tracks of coloured numbers following the processes of neurons through the stack of images, one coloured number for each different neuron (figure 4). If two tracks were found to coalesce at a branch point, one of the sets of coloured numbers had to be erased and replaced by the other. Finally, synapses had to be recorded and placed on neuron maps.

Eileen and John worked on several series Nichol had cut of adult hermaphrodites. None of these series covered the entire nervous system. But it was found in regions where the series overlapped that the cell positions and connections were so similar from animal to animal that corresponding cells in each series could be identified and a composite diagram put together. Meanwhile, Visiting Scientist Donna Albertson carried out the same process on other EM series through the somewhat independent pharyngeal nervous system and the posterior nervous system of the adult male. What was produced by this tracing method was not a full-scale three-dimensional rendering, but rather skeleton maps of each neuron. But this was sufficient to produce a formal connectivity diagram. The hermaphrodite diagram included about 5000 chemical synapses, 2000 neuromuscular junctions and 600 gap junctions. From John White's start in 1969 to completion, the project took 15 years.

**Figure 5.**
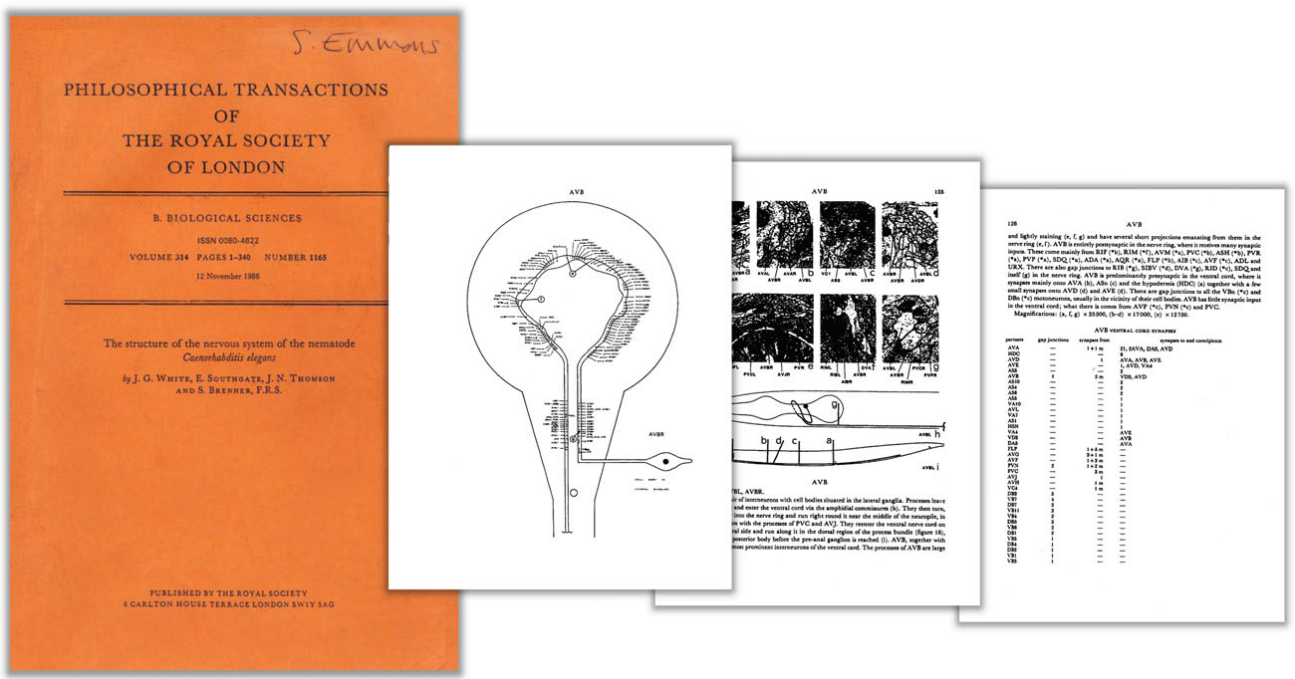
White *et al*. [1] present a map of each class of *C. elegans* neuron, together with electron micrographs of representative synapses, a description and lists of synaptic partners.

The manuscript sent to the Royal Society started with a section of introduction, methods and discussion of results, but the bulk of it was an appendix consisting of maps of each type of neuron in alphabetical order with associated representative electron micrographs of selected synapses and synapse partner lists (figure 5). There were altogether 132 such maps. On 5 September, Boycott sent Brenner his response. The paper would be accepted, although Boycott, who served as both editor and reviewer, had a considerable number of suggestions covering the introduction and discussion. (He did not concern himself with the maps—‘after all, few will read the detail’.)

But there was one big problem. At 450 pages, the manuscript was 350 pages over their maximum! It would cost the society above £17 000 to publish and would increase each subscriber's subscription cost by 25%! Eventually, funds were found and publication went ahead, but it had to wait a year and added a volume to the 1986 output. The authors were amused when they found the editors had used ‘The mind of a worm’ as the running head.

## 4. The origin of the nervous system

This paper was the last of a series from Brenner's group on the worm's connectome. Publications started with a description of the head sensory structures in 1975 [10], of the pharyngeal nervous system [11] and ventral nerve cord [8] in 1976 and of a partial reconstruction of the circuits in the male tail in 1980 [12]. (The male tail connectome was completed in the author's laboratory and published in 2012 [13].)

Important findings emerged right from the very first papers. One concerned a question in developmental biology—one of Brenner's two questions, ‘how is it built?’. At the same time that the EM reconstructions of the ventral nerve cord were coming out in the mid-1970s, another co-worker in the Brenner group, Staff Scientist John Sulston, was looking at cells. Using a light microscope fitted with the new Nomarski differential interference contrast optics, he had made the remarkable discovery that, observing through the worm's transparent cuticle, cell nuclei and their mitotic divisions could be seen in a living animal. Cell lineages could be traced by recording the series of cell divisions as a worm developed under a coverslip, feeding on a dot of bacteria.

Sulston confirmed what he had observed earlier with a staining method, that during larval development, a new set of cells was generated in the ventral nerve cord. At hatching, there were 15; a few hours later there were 57. This was surprising, as up to that time it was thought that cell division did not occur in nematodes after embryogenesis. Sulston found that the cells were produced by a 12-fold iteration of a stereotyped cell sublineage. The sublineage unfolded through four polarized cell divisions from a progenitor cell. The pattern of cell divisions was identical in every animal. John White's EM reconstruction showed that these new cells were motor neurons. A bet was placed regarding the relationship between the cell lineage and cell fate. John White won. He took Sulston's diagrams home over a weekend. By comparing the pattern of cells in the cell lineage diagrams to the pattern he knew of motor neurons arrayed along the ventral cord, he made an exciting discovery. With each iteration of the sublineage, a given branch gave rise to the same class of motor neuron. There was thus a correspondence between lineage ancestry and cell type. This result was published along with the EM reconstruction of the ventral cord [14].

By the early 1980s, Sulston and postdoctoral fellow Bob Horvitz, in Cambridge, and Judith Kimble, a graduate student at the University of Colorado in Boulder, were able to describe the entire cell lineage from the egg to the adult [15–17]. In 1983, at the famous international symposium held at the Cold Spring Harbor Laboratory that year on the topic ‘molecular neurobiology’, Sulston gave an account of the origin of every neuron in the nervous system [18].

The demonstration of an almost completely reproducible cell lineage opened up the question of how rigidly cell fates were determined by their cell lineage ancestry. The alternative was that a cell's fate is independent of its ancestry and is specified by signals in its local environment. This was a long-standing problem in the field of developmental biology. John Sulston investigated the question using a system developed by John White for focusing a laser beam through the objective lens of a microscope to kill cells. The idea was, if local signals were important, then cell fates might change if neighbouring cells were killed. Sulston found that in most instances, cells were not affected by killing their neighbours and the killed cells were not replaced. Thus most cell fates appeared to be inflexible and fixed at birth. But there were a few instances where cell fates were altered by the removal of their neighbours. It appeared that both ancestral specification and local signalling were at work in the worm, as they are now known to be in all animals [19].

## 5. Interpreting the map

A second important result to emerge from the first connectomics data concerned the functions of these same ventral cord motor neurons. They innervated the body wall muscles. Their structures immediately suggested how they worked: there was one set for forward locomotion, one set of opposite polarity for backward locomotion, a set of cross-inhibitors to reinforce an undulatory, swimming motion, as well as a set of command interneurons to control which group was operating. John White, John Sulston and postdoctoral fellow Marty Chalfie were able to verify these assignments by killing the various classes of cells with the laser [20]. Evidence for the functions of the motor neurons was also provided by studies of the 100-fold larger nematode, *Ascaris suum*, the intestinal parasite of pigs. This was studied by former Brenner colleague Tony Stretton at the University of Wisconsin. In spite of being 10 cm long, *Ascaris* was found to have clearly corresponding sets of motor neurons and command interneurons, but each one much bigger! In *Ascaris*, it was possible to show that the motor neurons were cholinergic and to verify by electrophysiological experiments that they drove the bodywall muscles, experiments not possible at that time for tiny *C. elegans* [21,22].

Not only were Sulston and Chalfie able to dissect the motor system, they were able to trace out an entire circuit for response to light touch. As seen with the motor neurons, the EM reconstructions don't just reveal the synaptic contacts of a neuron, they also make it possible to suggest its function from its structure, location and the identities of its synaptic partners. A set of neurons running underneath the cuticle and containing unusually large microtubules were candidates for touch receptors. Chalfie and Sulston verified this by killing them with the laser and showing that the worm lost sensitivity to a gentle stroke with an eyebrow hair [23]. Touch cells in the tail were wired into the circuit for forward locomotion and those in the head innervated the backwards circuit, reflecting the response of the worm, which is to move away from touch in these respective body regions. Thus, for some behaviours, the wiring diagram did indeed reveal how this nervous system might control behaviour, as Brenner had hoped. Moreover, this simple touch response could be readily dissected genetically and yielded a rich set of mutants with defective touch cells, identifying just the sort of genes Brenner had in mind, genes that specified the structure as well as the function of the nervous system [23,24].

For other behaviours, however, identifying circuits turned out not to be so straightforward. Motivated by the available wiring diagram and the large collection of uncoordinated mutants, a number of laboratories set about learning in greater detail what a worm could do besides respond to light touch. Not surprisingly, worms endeavour to do all the same things other animals do: locate food, stay safe, reproduce and disperse. Worms find food by chemotaxing up chemical gradients of attractive cues emitted by bacteria. They eat by pumping bacteria into their pharynx and gut. If they lose track of their food source, they try to re-find it by returning to the conditions of salt and temperature where they last were on it. Thus they can learn this association and they can remember it. They attempt to stay safe by moving away from noxious chemicals or harmful osmotic conditions. They avoid solutions that previously contained predatory nematodes. Reproduction involves an egg-laying programme on the part of the hermaphrodite and a complex copulatory behaviour on the part of the male (figure 1). If males are separated from mates, they will explore away from a food source to find them. When food runs out, some larval worms develop as the resistant dauer form first sectioned by Nichol, which, in hopes of being picked up by a passing invertebrate, stands on its tail and waves its head about. As many of these behaviours are mutually exclusive, worms face the decision-making task of deciding, from moment-to-moment, what would be the most advantageous thing to do.

**Figure 6.**
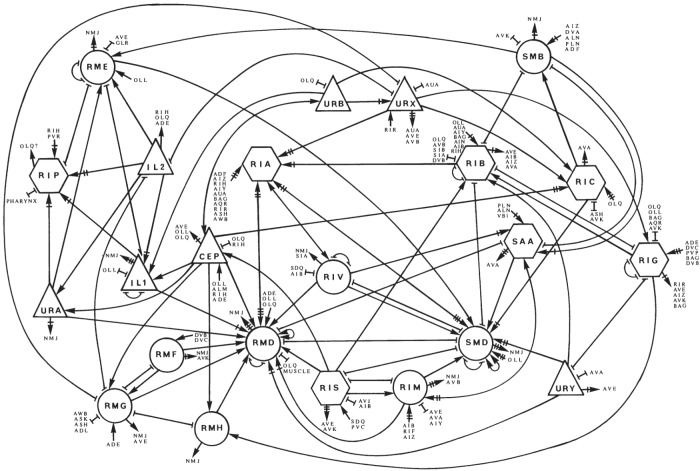
Diagram of the connections between neurons that primarily lead to head muscles, from ‘The mind of a worm’ [1, fig. 21c]. Triangles represent sensory neurons, hexagons are interneurons and circles are motor neurons. Lines with arrowheads represent the directions of chemical connections; whether these are excitatory or inhibitory cannot be determined from the electron micrographs. Cross lines behind arrowheads indicate relative strength of connection. Lines ending in bars are connections created by gap junctions. Each connection is made by one or more synapses. Connections to additional neurons are given in the lists. Copyright © The Royal Society.

Most of these behaviours are controlled by circuits in the nerve ring and anterior ganglia (figure 2). Pumping is additionally controlled by a nearly isolated set of neurons within the pharynx, while male mating is controlled by the circuitry in the male tail. In the anterior ganglia and nerve ring, in contrast to the ventral nerve cord which drives locomotion, no clear circuits were apparent in the wiring diagrams. To be sure, a general flow of information from sensory inputs through other neurons to muscles was evident [1,25]. But the neurons in general were so heavily cross-connected by chemical and gap junction synapses that the result was a network in which one could traverse from any neuron to nearly any other one in just a few steps (figure 6).

Deciphering how this network of neurons controls behaviour has awaited the progress of much further research and is ongoing. For a start, it was necessary to describe the behaviours in finer detail. Movement up or down gradients of chemicals is governed in part by behaviour properly described as a kinesis―biased movement based on the ratio of ‘runs' and ‘turns' [26]. Improving conditions promote runs, worsening conditions promote turns. While the overall outcome of this behavioural pattern is predictable—movement towards favourable conditions and away from unfavourable ones—the precise moment when a turn will be executed is not. The turn event occurs in what appears to be a stochastic manner described by a frequency or probability. How a network of neurons generates this type of behavioural output is currently the subject of great interest.

In other invertebrate systems where a network of identifiable neurons governs a relatively simple behaviour, such as the gill-withdrawal reflex circuit of the sea hare *Aplysia californica* or the stomatogastric ganglion of crustaceans, the method of choice to approach such a problem is electrophysiology. In this method, the function of neurons in a circuit during behaviour is assessed or controlled by inserting pipette needles into them. But *C. elegans* neurons are too small and this method has only become possible recently and remains difficult to apply [27]. The breakthrough has come through molecular genetics. Now transgenes whose products fluoresce with Ca^++^ concentration or voltage, or that open and close channels with light stimulation, allow *C. elegans* neuron activities to be observed and controlled almost at will.

The principles of *C. elegans* nervous system function are now emerging in a large number of papers taking advantage of these techniques. Ongoing studies of mutants continually identify critical molecular components. Important among these are neurohormones, which allow neurons to communicate extrasynaptically over large distances [28–30]. It has become possible to work towards fully reverse engineering the network and developing quantitative models that predict its output over time. As the nervous systems of probably all animals are similarly constructed neural networks, the results in *C. elegans* are expected to have broad applicability.

## 6. Genetic underpinnings

As soon as he had behavioural mutants and interpretable electron micrographs, in the late 1960s, Brenner began to look for genetically encoded structural changes in the nervous system. And he found them. By the time John White joined the project, Brenner had already discovered three mutants with abnormalities of motor neurons [4,6]. Other members of the laboratory quickly joined in the search. ‘We behaved like kids in a candy store’ (J. G. White 2014, personal communication to S.W.E). Asking Nichol to section and image some 30 ‘uncoordinated’ mutants selected for their intriguing behavioural abnormalities, they examined the images hoping to find interesting things.

Five of the genes were found to have clear defects in the outgrowth and wiring of motor neurons. Sabbatical visitor Bob Wyman picked up two of Brenner's mutants, both in the gene *unc-5*, and confirmed Brenner's observation that there was no dorsal nerve cord [31]. John White explored further the third mutant (*unc-30*) and two others (*unc-3* and *unc-4*), while postdoctoral fellow Leon Nawrocki traced another (*unc-55*). Additional studies analysed the neurons generated in lineage mutants (*lin-5*, *unc-59* and *unc-85*) and the ‘undead’ cells in cell death mutants (*ced-3* and *ced-4*). A survey of 19 chemotaxis-defective mutants revealed many to have disorganized head sensory structures. All these results immediately vindicated Brenner's vision, that by identifying mutants and then looking for their effects on the nervous system, genes could be found that specified nervous system structure.

Alas, it was easier to make these observations than to interpret them. For each mutant, because of the large amount of effort involved, only one or a few individuals could be examined. There was often considerable disorganization, making interpretation dicey―what was the primary defect? Wyman found it frustrating that only the static EM results were available, with no way to assess neural activities. One could imagine many ways development of the dorsal nerve cord could be blocked. No clues could be obtained from the gene products, which were unknown. There was an ethos in the MRC-LMB laboratory that one did not publish until one had a complete story about an interesting problem. And moreover, there was limited pressure to publish papers. The laboratory was protected by a hands-off policy of the MRC governing board that allowed work to go forward without periodic review. Two papers were submitted to Nature (*unc-30* and *unc-55*) but were rejected and never resubmitted elsewhere. Only the results with the lineage and cell death mutants and the disorganized sensory structures were published at the time [32–35].

Publication of the mutants with structural and synaptic changes has taken many years and, like interpretation of network function, awaited many developments. One was the ability to visualize neuron processes in the light microscope. Using a permeable fluorescent dye, it was possible to see the neurons in many worms, overcoming the barrier of small sample size [36,37]. With this and additional methods (e.g. antibody and lectin staining), Ed Hedgecock and co-workers showed that *unc-5* was one of three genes, along with *unc-6* and *unc-40*, that constitute a topographical signalling system in the body. This system is present in all animals. The protein product of the vertebrate version of *unc-6* was discovered independently in studies of the vertebrate spinal cord and named Netrin, while the mammalian version of *unc-40* had been found as a gene deleted in colorectal cancer and accordingly named DCC. But it was in *C. elegans* that all three were first shown, in a 1990 paper, to act together as a signalling system for organizing the nervous system and other body structures [38].

The approach of fluorescent cell labelling became a universally applied, enormously powerful technique in *C. elegans* and subsequently throughout biology with the introduction of green fluorescent protein, GFP. The utility of GFP for labelling cells was demonstrated by Marty Chalfie, now at Columbia, in 1994 [39]. Chalfie first heard about this marvellous jellyfish product in a seminar in 1989 [40]. He instantly recognized its potential for *C. elegans* research. The worm is transparent, so its internal structure is visible. Several methods had been developed for visualizing protein or RNA expression in single cells, but they were difficult, destructive techniques. GFP, which absorbs UV or blue light and emits green light, held out the promise of making gene expression readily observable in a living worm. Individual cells could be labelled by constructing a transgenic worm that expressed the GFP gene under the control of a cell-specific chromosomal regulatory sequence. Following his publication, Chalfie was inundated with requests for the gene from biologists all over.

Not only does the expression of GFP in a particular cell reveal that the chromosomal sequence driving it was activated in that cell, the small, diffusible GFP protein monomer fills the cytoplasm and reveals the shape of the cell. In transparent *C. elegans*, the processes of every neuron became visible (figure 2). This allowed verification of the EM maps created by John and Eileen, resulting in the discovery of just a single tracing error (just *one*!) (D. Hall and O. Hobert 1999, personal communication). With neural processes revealed in living worms, mutations affecting process outgrowth and placement could be readily isolated. Moreover, GFP could be joined to cellular proteins to track their locations. With this approach, individual synapses and neural connections could be fluorescently labelled, making isolation of connectivity mutants possible [41,42].

With the introduction of molecular cloning during the 1970s and 1980s, the protein products of genes could finally be identified and their expression patterns determined. This information allowed suggestions to be made about the functions of the genes that affected nervous system structure and helped to understand the defects seen in mutants. One of the genes with an interesting behavioural phenotype studied earlier by John White, *unc-4*, was cloned by David Miller, a former postdoctoral fellow at the MRC-LMB now working at Vanderbilt University. *unc-4* worms could swim forward normally, but when touched on the head, they could not reverse and swim backwards. Instead, they curled up. John White showed that motor neurons had normal projections but were miss-wired. Consistent with the phenotype, the set of motor neurons for backwards locomotion on the ventral side lacked their normal inputs from the backwards command interneurons and instead received input from the forward command interneurons. Thus, the backwards motor neurons on the ventral side had become wired like the forward ones. Miller found that *unc-4* encoded a transcription factor, a type of nuclear, DNA-binding protein that regulates gene expression. This transcription factor apparently regulated genes encoding functions that determined the wiring specificity of the motor neurons. With this mechanistic insight in hand, the EM results were published alongside the molecular interpretation in 1992 [43,44]. Along with a similar amount of additional information, the wiring changes in *unc-30* and *unc-55* are just now being published [45]. Like *unc-*4, each of the genes *unc-30*, *unc-55* and *unc-3* encodes transcription factors [46].

We now know the protein products of all 30 ‘uncoordinated’ genes studied in the initial EM investigations. Eight are components of synapses. Another 11 are proteins found in all cells involved in various aspects of cell structure and function. These results reflect the unanticipated contribution *C. elegans* research has made to cell biology in general and cell biology of the nervous system in particular.

The set that gave clear changes to nervous system structure includes, in addition to *unc-5* and two genes that work with it, eight transcription factors. This hints that a change in the expression of multiple genes is required to bring about rewiring or loss of connectivity. This may explain the surprising absence of a class of genes anticipated by Brenner: genes encoding proteins that function as molecular cell labels to allow pre- and post-synaptic cells to recognize each other. This event is crucial for establishing a wiring diagram. Molecular cell labels should consist of secreted or transmembrane proteins with extracellular protein–protein interaction domains. There are many such genes in the *C. elegans* genome, as in the genomes of all animals. Deliberate genetic searches have identified a few that are involved in synapse formation [41,42]. But there must be a very large number responsible for the complex pattern of connections. Why more mutations in them have not been found remains to be seen.

Methods for identifying the protein products of genes and their expression patterns in tissues were needed for progress in every branch of biology. Developing this capability became the agenda for the next decades in many laboratories, beginning with the discovery of nucleic acid enzymes that could be used to manipulate DNA, cutting it at defined sites and joining pieces together, the purification of biochemical quantities of individual genes through cloning them in bacterial cells, through DNA sequencing and ultimately sequencing the human genome itself. By the early 1970s Brenner turned his attention to development of these methods.

## 7. The new field of *C. elegans* research

Spearheaded largely by Brenner's co-workers, postdoctoral and sabbatical fellows, the field of *C. elegans* research began to grow. Many recruits, like Brenner himself, moved to *C. elegans* from prior work on bacteria and bacterial viruses, attracted by the powerful genetics and simple structure of the worm. Some laboratories continued to focus on the nervous system. For their work, ‘The mind of a worm’ was the Bible. It has been cited over 2500 times, a number currently increasing at the steady rate of 0.82 citations per day. Others looked at mutants that affected development. The discovery that purified genes could be easily introduced into worms by simply microinjecting them was a key advance [47,48]. The drive to identify the molecular products of *C. elegans* genes known by their mutant phenotypes led to the first physical chromosomal map correlated with a genetic map of a multicellular organism, followed by the first such genome sequence [49,50]. Genome sequencing, regulatory systems such as *unc-5*/*unc-6*/*unc-40*, microRNAs (miRNAs), RNA interference (RNAi, Nobel Prize 2006), programmed cell death (Nobel Prize 2002), GFP (Nobel Prize 2008), are just the beginning of a long list of contributions from a field today consisting of over 1000 laboratories worldwide. The desire to observe more clearly the structure of the *C. elegans* embryo led John White to develop the first practical confocal microscope [51]. The results of *C. elegans* research have been periodically summarized in two published books, one in 1988 and one in 1997, and now in a continually updated ebook [52,53], WormBook.org.

Research on *C. elegans* has three important attributes. The first is that it is founded on observational, descriptive science. Ever since Rutherford said ‘all science is either physics or stamp collecting’, there has been a prejudice in favour of experimental, so-called ‘hypothesis-driven’, ‘hard’ science. But of course you first have to know about something before you can formulate a hypothesis about it. Brenner called it ‘have a look science’. Descriptive observation is in fact at the core of scientific investigation.

The second attribute is one of completeness―the description needs to be complete. Brenner emphasized this point when proposing to determine the wiring diagram of an animal *in its entirety*. He is fond of pointing out the necessity of refuting the sceptic who says there is another wire. ‘There are no other wires, we know *all* the wires!’ In addition to the connectome, the cell lineage represents a second set of complete observations underlying *C. elegans* research. And of course the genome sequence is a third, considered a landmark in biology. Such completeness allows a greater degree of rigor when drawing conclusions from experiments. Possibly the success of *C. elegans* research has led to a more accommodating attitude towards this approach today.

Finally, the field of *C. elegans* research embraced the open sharing of information and materials between laboratories. A philosophy that a rising tide raises all boats was promoted by the Cambridge MRC-LMB workers, who freely sent out before publication their findings on the cell lineage or nervous system connectivity, mutant worm strains, and later, DNA clones. To assemble the genome sequence, the entire community was mobilized to contribute their DNA and genetic mapping results. Communication was fostered by a quarterly Worm Breeder's Gazette, started by Robert Edgar of the University of California. Here, the latest results were circulated to all. The short articles were to be treated as personal communications and never cited without permission. A biennial international meeting was started in 1977, where, in an always exciting atmosphere, everyone shared what they knew. The talks were given almost exclusively by the graduate students and postdoctoral fellows who did the work. Led by John Sulston, the philosophy of freely and rapidly sharing valuable, publicly funded findings eventually was adopted by the human genome sequencing consortium [54]. It has become a more widely accepted practice today.

## 8. Returning to connectomics

By the end of the twentieth century, computers had gained sufficient power to lend themselves to nervous system reconstruction in the way that Brenner had envisioned nearly 40 years before. In 1999, this writer and colleagues began to think about the unfinished male tail reconstruction begun by Donna Albertson in the 1970s. By digitizing Nichol Thomson's electron micrographs, already almost completely annotated by Donna with the Rapidograph pens, and employing software designed to allow the annotation to be carried out with mouse clicks at the computer screen, they were able to complete the reconstruction [13]. The result was astonishingly complex—the neural network for male mating nearly doubled the number of synapses in the nervous system. What had taken over a decade before now took only two years once the software was in place. By this time, other scientists had also begun to face the problem of nervous system ultrastructure. Viewing the enormous effort that had been required to obtain the first *C. elegans* connectome, in the intervening years no one had made any further attempts. But with the prospect of computer assistance, several laboratories began to address the multiple technical challenges—the tedious and treacherous step of creating long, unbroken series of sections; the slow image acquisition by EM; the difficulty of annotating the complex images; and finally analysis of the data [55–57].

Even advances in the mathematics of networks lent themselves to the problem. The software used to generate the *C. elegans* male posterior connectome allowed a quantitative measure of the size of each synaptic connection, a proxy for synaptic strength. With this quantitative description, it was exciting to discover that using techniques from the mathematical field of graph theory it was possible to find functional sub-circuits within the network [13]. The static structure alone, when analysed in the right way, was interpretable.

## 9. Ideas evolve

One reason it has taken so long to fully exploit or understand the 1986 description of the *C. elegans* nervous system was that enormous gaps in our knowledge of genes and cells had to be filled in first. But a second reason ‘The mind of a worm’ is gaining greater appreciation today has to do with how the nervous system was and is viewed.

In his 1973 article describing his ideas for how to understand the nervous system, Brenner emphasized its genetic underpinnings and did not discuss learning and memory. When Bob Horvitz suggested studying learning and memory for a postdoctoral project, Brenner replied that he did not think worms learned or remembered anything! The field of neuroscience, on the other hand, descended as it was from physiologists and psychiatrists, from the time of Hebb maintained a focus on learning and memory [58]. Where Brenner's approach was genetics, theirs was electrophysiology. Brenner called them ‘the electricians' [5]. The two approaches required different experimental animals―geneticists required little ones, electrophysiologists required big ones, or at least ones with big cells. Neuroscientists thought the nervous system could be understood by understanding the electrical properties of neurons and the plasticity of synapses. In their view, learning and memory were the nervous system's essential and indispensable features. If connectivity mattered, it was thought, possibly it could self-assemble. As for EM, a 1988 perspective article does not list this among the methods available for studying the nervous system [59].

When John White presented an analysis of the *C. elegans* wiring diagram at the same 1983 Cold Spring Harbor Symposium where John Sulston presented the cell lineage of the nervous system, he felt it ‘went down like a lead balloon’ [60]. Two years earlier at a meeting of vertebrate neurobiologists he perceived his talk was met with such indifference he gave his free meal tickets to a hungry looking student and went home early (J. G. White 2014, personal communication to S.W.E). The difference of perspective illustrated by these reactions—the gulf between the geneticists and the neuroscientists—embodies the hoary question of nature versus nurture, a question that runs as an enduring storyline throughout intellectual history [61].

Now an evolution, if not a revolution, in thinking has come about. The change of view has emerged in part from the recognition of the unity of life revealed by genomic sequences. The *C. elegans* nervous system contains many of the same molecules as the human nervous system [62]. The common ancestor of worms and humans already had a complex nervous system and behaviour. Further impetus to a change in thinking has come from finding that genetic causes underlie some severe mental disorders―diseases of the ‘mind,’ like schizophrenia and autism. Some of these disorders may in fact be connectopathies caused by mutations.

In the end, it's all coming together now, genetics and physiology. As a measure of how far we have come, whereas the White *et al.*'s paper [1] in 1986 was initially met with indifference outside the community of *C. elegans* researchers, the 2012 paper on the posterior connectome of the *C. elegans* male [13] received the prize awarded each year by the American Association for the Advancement of Science for the most outstanding research article published in its journal *Science*. Recently, a *C. elegans* researcher was appointed co-chair of a United States government initiative to study the brain and was chosen as a co-organizer when the topic of the Cold Spring Harbor Symposium in 2014 was *cognition*.

Yet in spite of this progress, the piece of the puzzle identified by Brenner, the detailed synaptic connectivity of the nervous system, remains today unknown for larger animals. The development of new technologies and methodologies in the field of connectomics offers an enormously exciting prospect. New connectomes will open up a window into what previously has been a black box of biology: the comparative ultrastructural anatomy of nervous systems. How will connectivity in the brain of a fruit fly with 100 000 neurons, or in the human brain, 10^8^ times larger than *C. elegans*, compare? Will the nervous system of *C. elegans* prove to closely resemble the ancestral condition of all nervous systems and serve as a relevant model? Or is its relatively simple and fixed structure indicative of a highly derived form?

A connectome reveals the structural features that underlie nervous system function. As with a genome sequence, its value is not that it provides an answer to every question (although it provides the answers to some), but that it serves as the basis for formulating questions that need to be answered to understand function. The 1986 paper dubbed ‘The mind of a worm’ has served this function well for research on *C. elegans* behaviour. Beyond this, the paper was of enormous overall significance in promoting research in all areas of what became the new field of *C. elegans* research. This field has had an important influence on the way biological research is carried out today. Over a period of 50 years, it has, directly and indirectly, made an outsized contribution to biological discovery and understanding.

## Author profile

**Scott Emmons** is the Siegfried Ullmann Professor of Genetics and Professor of Neuroscience at the Albert Einstein College of Medicine. His broad research interest is in how the genes of an organism determine its phenotype. Using the nematode *C. elegans*, his laboratory has made contributions in the areas of genome structure, including transposable elements, genetic specification and evolution of morphology, specification of cell fate during development, sex determination, nervous system development, behaviour, and most recently, connectomics. His 2012 paper on the connectome of the posterior nervous system of the *C. elegans* male won the Newcomb Cleveland Prize for most outstanding research article in the journal *Science*. Dr Emmons is a graduate of Harvard College and received his PhD from Stanford University in 1974.
